# Glymphatic-lymphatic coupling: assessment of the evidence from magnetic resonance imaging of humans

**DOI:** 10.1007/s00018-024-05141-2

**Published:** 2024-03-13

**Authors:** Geir Ringstad, Per Kristian Eide

**Affiliations:** 1 Department of Radiology, Oslo University Hospital - Rikshospitalet, Oslo, Norway; 2 Department of Geriatrics and Internal Medicine, Sorlandet Hospital, Arendal, Norway; 3 Department of Neurosurgery, Oslo University Hospital - Rikshospitalet, Oslo, Norway; 4 Institute of Clinical Medicine, Faculty of Medicine, University of Oslo, Oslo, Norway

**Keywords:** Glymphatic system, Brain-immune cross-talk, Brain clearance, Contrast media, Non-invasive imaging

## Abstract

The discoveries that cerebrospinal fluid participates in metabolic perivascular exchange with the brain and further drains solutes to meningeal lymphatic vessels have sparked a tremendous interest in translating these seminal findings from animals to humans. A potential two-way coupling between the brain extra-vascular compartment and the peripheral immune system has implications that exceed those concerning neurodegenerative diseases, but also imply that the central nervous system has pushed its immunological borders toward the periphery, where cross-talk mediated by cerebrospinal fluid may play a role in a range of neoplastic and immunological diseases. Due to its non-invasive approach, magnetic resonance imaging has typically been the preferred methodology in attempts to image the glymphatic system and meningeal lymphatics in humans. Even if flourishing, the research field is still in its cradle, and interpretations of imaging findings that topographically associate with reports from animals have yet seemed to downplay the presence of previously described anatomical constituents, particularly in the dura. In this brief review, we illuminate these challenges and assess the evidence for a glymphatic-lymphatic coupling. Finally, we provide a new perspective on how human brain and meningeal clearance function may possibly be measured in future.

## Background

The study of lymphatic vessels in dura mater has a long history, but in recent time, this was facilitated by the introduction of specific markers for lymphatic endothelial cells [1]. In 2015, animal studies unveiled for the first time that tracer in cerebrospinal fluid (CSF) drains directly into lymphatic vessels within the dura, primarily alongside the dural venous sinuses [2, 3]. It was furthermore demonstrated that meningeal lymphatic drainage assumes a pivotal role not only in eliminating solutes from the subarachnoid space (SAS), but also from the brain itself, implying that brain solute clearance along perivascular pathways is dependent on meningeal lymphatic vasculature. As such, later studies showed that experimental ablation of meningeal lymphatic drainage was followed by delayed clearance of both tau [4] and amyloid-beta [5] from the animal brain.

In this review, we provide as background a short summary of current, co-existing concepts for brain perivascular clearance, including the glymphatic system, which currently seems to have the largest momentum. Furthermore, we describe shortly main elements of the breakthrough research findings of dural lymphatic anatomy and function in rodents. In humans, we identify previous anatomical research on dura, particularly the parasagittal dura and its constituents. From this, we critically illuminate translational magnetic resonance imaging (MRI) investigations in humans that have endeavored to image the intricate interplay between brain perivascular drainage and lymphatic drainage from the craniospinal compartment. We particularly scrutinize potential pitfalls in the interpretation of applied MRI studies within this emerging research field. Finally, we recommend a new method to assess meningeal CSF clearance capacity in research and the clinic.

### The glymphatic system and competing models

**Fig. 1:**
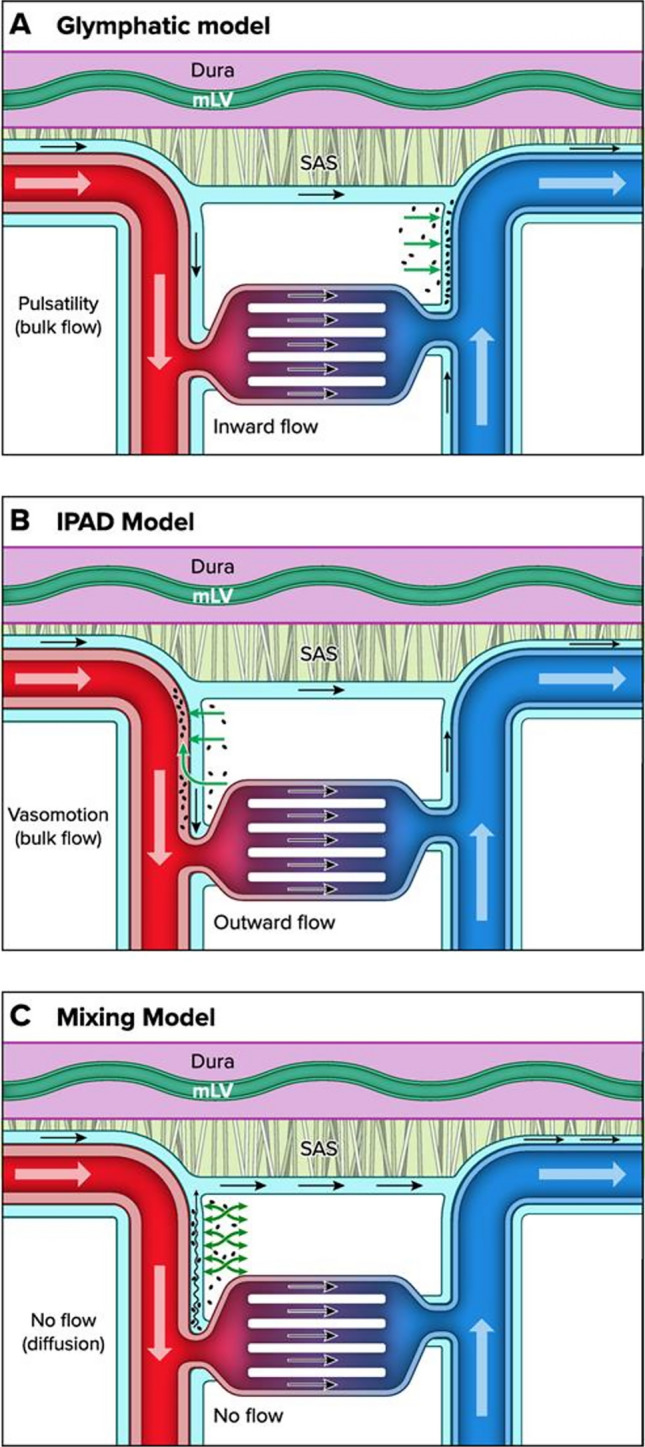
Competing model frameworks for understanding perivascular clearance. All models currently accept CSF influx along penetrating arteries and mixing with the interstitial fluid, but drainage routes from brain differ substantially. In the glymphatic system (**A**), interstitial solutes are cleared along veins into CSF or directly to meningeal lymphatic vessels, in the IPAD model (**B**) drainage is within the artery wall, and in the mixing model (**C**), solutes diffuse along a concentration gradient along periarterial spaces back into CSF, facilitated by mixing from physiological motion. Illustration from Zhao et al. (Physiology) [14] (reuse by permission from CCC RightsLink® service)

The initial elucidation of the glymphatic system in rodents [6] has significantly influenced imaging of human brain clearance along perivascular pathways over the past decade, fostering a burgeoning realm of translational research [7]. One of the main breakthrough observations in humans was that exchange between cerebrospinal fluid (CSF) and brain tissue is extensive and profound, and clearance from both CSF and brain differs between individuals as well as between patient groups, including those with idiopathic normal pressure hydrocephalus (iNPH)-associated dementia [8, 9]. Nevertheless, some unresolved inquiries remain. Presently, the extent to which perivascular solute clearance contributes to overall brain clearance remains ambiguous when juxtaposed with other well-established pathways, including solute excretion across the blood–brain-barrier (BBB), local proteolytic cleavage, and microglial resorption [10]. The role of impaired glymphatic function has been postulated as a causal factor and consequence within a spectrum of neurological disorders [11, 12]. However, the glymphatic paradigm faces direct contradictions from researchers attempting to reproduce the experimental framework [13] and is challenged by alternative hypotheses for perivascular clearance, such as intramural periarterial drainage (IPAD) and periarterial mixing [10, 14] (Fig. 1). Despite these variances, all competing frameworks now seem to converge on the consensus that the influx of cerebrospinal fluid (CSF) from the brain surface serves as the principal propellant within these pseudo-lymphatic clearance systems. In previous reports of the IPAD system, CSF influx along arteries was not supported, based on the belief that cortex lacked perivascular spaces [15], but this view seem now to have been abandoned. A shared characteristic is further the scarcity of evidence for a direct anatomical connection between the brain and lymphatic vessels at the meninges and, ultimately, the bloodstream.

In the primary conceptualization of the glymphatic system, the question of additional drainage routes from brain, whether directly into the subarachnoid space (SAS) or traversing cortical veins to eventual lymphatic vessels remained open. In the IPAD model [16], meningeal lymphatic vessels are by-passed, as solute drainage within the arterial wall muscle layer is supposed to persist uninterrupted and travel opposite of the blood stream within the carotid artery wall directly to cervical lymph nodes, albeit without direct evidence of such connections. Conversely, periarterial mixing entails solutes diffusing from the brain interstitium along their concentration gradient directly into periarterial spaces, where pulsations facilitate the retrograde pumping of solutes back into the SAS.

Curiously, if the influx of CSF into the brain is of paramount importance, the notion that brain waste is cleared back into CSF at the surface as to a common sink, and potentially recirculated into the brain, might appear counterintuitive. Therefore, the anticipation that brain perivascular drainage connects directly with meningeal lymphatic vessels has supposedly influenced interpretation of imaging findings from the human setting.

### Imaging of meningeal lymphatic structures and their functional characteristics in rodents

In their seminal study from 2015, Louveau and coworkers detected that fluorescent CSF tracer drained into vessels adjacent to the sagittal and transverse dural sinuses (dorsal regions) that were positive for several lymphatic endothelial cell markers [2]. These vessels were considered to represent initial lymphatic vessels, covering less of the tissue and forming a less complex network composed of narrow vessels compared to other body regions. Diameter of the vessels was shown to be at micrometer scale, and it was hypothesized that the vessels may add to CSF filtration via arachnoid granulations, which are prominent in the perisinous region. Finally, it was proposed that meningeal lymphatic vessels may represent the second step in the drainage of interstitial fluid from the brain parenchyma after primary drainage into CSF. In the same year, Aspelund et al. [3] in another seminal study observed that the dye they injected into brain parenchyma drained into CSF along paravenous routes and entered the CSF space before drainage to deep cervical lymph nodes, primarily via lymphatic vessels that were exiting through foramina at the skull base. In later studies, lymphatic vessels were reported to be the major outflow pathway for CSF [17] and that ventral meningeal lymphatic vessels at the skull base were the hotspots for lymphatic drainage of CSF, not those in the dorsal perisinus region [18]. It was more recently demonstrated that the parasagittal dural stroma is rather an important hub for brain-immune cross-talk, where circulating immune cells can enter its stroma directly from the venous sinuses and here be presented to brain specific antigens by local dendritic cells [19]. Moreover, arachnoid granulations have been shown to communicate with this stroma and also to harbor immune cells, suggesting these trans-arachnoid passage ways may also represent lymphatic conduits with a neuroimmune role [20].

### The human parasagittal dura

Rodent and human brain differ in many aspects, not only in the most obvious ways concerning size and gyration patterns; but also clearance of tracer from CSF occurs of a time scale of days in humans [8], compared to a few hours in rats [21], with the pig brain comparing closer to the human [22]. Functionally, it has also been provided evidence that CSF lymphatic efflux routes may differ; while nasal lymphatic efflux is consistently shown to be of major importance in rodents [23], an in vivo CSF tracer study in humans detected no efflux through the cribriform plate to nasal mucosa [24], which contradicts previous non-MRI studies [25, 26]. There may also be species differences regarding impact of sleep and circadian rhythms on meningeal clearance function. Hence, lymphatic efflux of CSF tracer examined by near-infrared fluorescence imaging, was faster in awake than anesthetized mice [27]. In humans, on the other hand, there was in one patient cohort no effect of one night`s sleep deprivation on efflux of CSF tracer to parasagittal dura [28]. In the brain, however, CSF tracer dynamics was affected by both acute sleep deprivation [29] and chronic sleep impairment [30]. Further translational research in humans therefore seem mandated.

Fox et al. performed in 1996 a detailed anatomical study of the parasagittal dura based on formalin-fixed human autopsy specimens. In addition to intradural arachnoid granulations, they reproduced presence of an extensive network of intradural, endothelial-lined channels stemming from the superior sagittal sinus along nearly its entire length. These channels were first described by Weed in 1917 [31], and has later been demonstrated by others [32–34]. Fox et al. assessed the dimensions of intradural channels, which ranged from 0.02 to 2.0 mm and either connected to the superior sagittal sinus or lateral lacunae, which extended up to 3 cm from midline. The network of intradural channels was unrelated to cortical veins and was proposed to represent a pathway for the flow of CSF from arachnoid granulations to the superior sagittal sinus. Meningeal veins also appeared distinct from the intradural channels, but not branches of meningeal veins, which arborized medially and to some extent connected there with intradural channels. Intradural channels have a superior location to arachnoid granulations [32], which may imply their role in trans-dural CSF efflux to skull bone marrow [35]. A review by Mack et al. [36] also emphasized the extensive venous network within dura, as well as the meningeal arteries and accompanying meningeal veins, which are superficially located in the outer aspect of dura, and finally capillaries, that are present throughout the dura and are extremely rich in the parasagittal region. Dural lymphatic vessels were identified to be at micrometer scale in the parasagittal dura from histological analysis of whole-mount human specimens [37]. Recently, dural lymphatic vessels were also demonstrated utilizing immunohistochemistry in specimens retrieved from patients [1, 38]; the lymphatic vessels were seen in specimens from patients and obtained about 1.5 cm lateral to the parasagittal dura, and also in more remote locations. Here, lymphatic vessels were both localized nearby blood vessels as well as in distance to blood vessels. Given the methodological challenges with identifying lymphatic vessels using formalin-fixed tissues [1], it remains to be determined the relationship between intradural channels in parasagittal dura and lymphatic vessels.

### Attempts to visualize meningeal lymphatics in parasagittal dura with MRI

Regarding imaging of human meningeal lymphatics, MRI has typically been the preferred methodology due to its non-invasive nature, and has mainly focused on the parasagittal dural region. Various MRI based approaches have been performed, by both assessing enhancement patterns of MRI contrast agents administered intravenously and intrathecally, but also without any use of contrast agent. In any case, since blood vessels, intradural arachnoid granulations and intradural channels are abundant in the parasagittal dura, these need to be accounted for in attempts to visualize lymphatic vessels in this region. Accordingly, a commonly acknowledged challenge with MRI of lymphatic vessels in other body regions (MR lymphangiography) has been to separate contrast enhancing blood vessels from lymphatic vessels. For superficial body lymphatic vessels, this has been accomplished by obtaining MRI after injecting contrast agent into the cutis to visualize lymphatic channels through a 30 min period, and subsequently, an MRI 2–3 min after intravenous contrast to define veins [39]. Lymphatic vessels in limbs will typically show progressive contrast enhancement with the greatest degree after 30 min and appear with a characteristic irregular, beaded pattern. Veins have typically a smooth, uniform caliber and are visible 2–3 min after intravenous injection. MRI contrast agents have typically a half-life in blood of approximately two hours, are therefore present within the blood circulation and thus also in peripheral tissue for several hours [40].

**Fig. 2:**
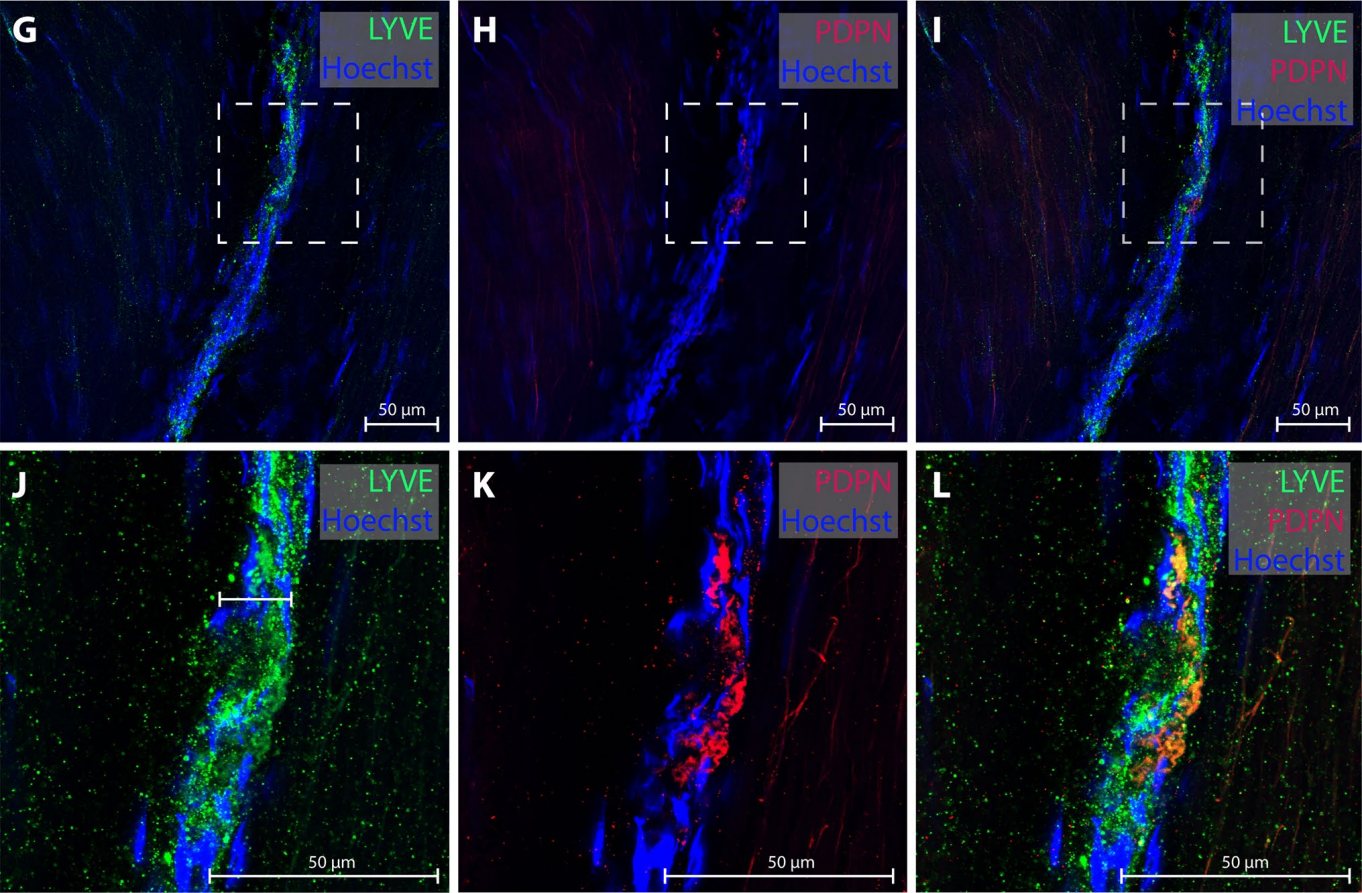
Human dural lymphatic vessel from the upper frontal convexity. Co-localization of the lymphatic markers lymphatic vessel endothelial hyaluronan receptor 1 (LYVE-1) and podoplanin (PDPN) confirms the presence of meningeal lymphatic vessels in dura mater of patients (panels **J**–**L** are magnification of panels **G**–**I**). The vessels have a diameter at micrometer scale and would not be expected to be delineated at mm-scale MRI. Courtesy of Fig. 7 **G**–**L** from Quesada et al. FBCNS) [1], reprinted under the terms of the Creative Commons Attribution License (CC BY)

Several reports of MRI studies claim to have directly visualized dural lymphatic vessels after administration of intravenous contrast [41–47], some with addition of the precautious prefix “putative” [47]. Typically, single, mm-thick tubular structures are demonstrated in 2D or 3D and conclusions about their lymphatic origin typically rely on a topography fitting with descriptions of dural lymphatic networks in rodents or neuropathological data. Strikingly, there are to our knowledge no studies discussing the possibility that tubular shaped structures at MRI might represent veins or derive from other known, abundant constituents in the region. There is also broadly a lack of considerations that vessels visualized at MRI are orders of magnitude larger in diameter than what has been described in animal [2, 3, 19] as well as in human [1, 38, 48] studies. In one human study, lymphatic vessel diameter at various location remote from sinus veins varied from approximately 20–40 µm [38] (Fig. 2), and in another, meningeal lymphatic vessels adjacent to the superior sagittal sinus ranged from 8 to 119 µm [48]. Thus, lymphatic vessels at MRI should not be expected to have diameter larger than ~ 0.1 mm, and often even less, which is far below the typical MR image resolution (~ 1 mm).

**Fig. 3:**
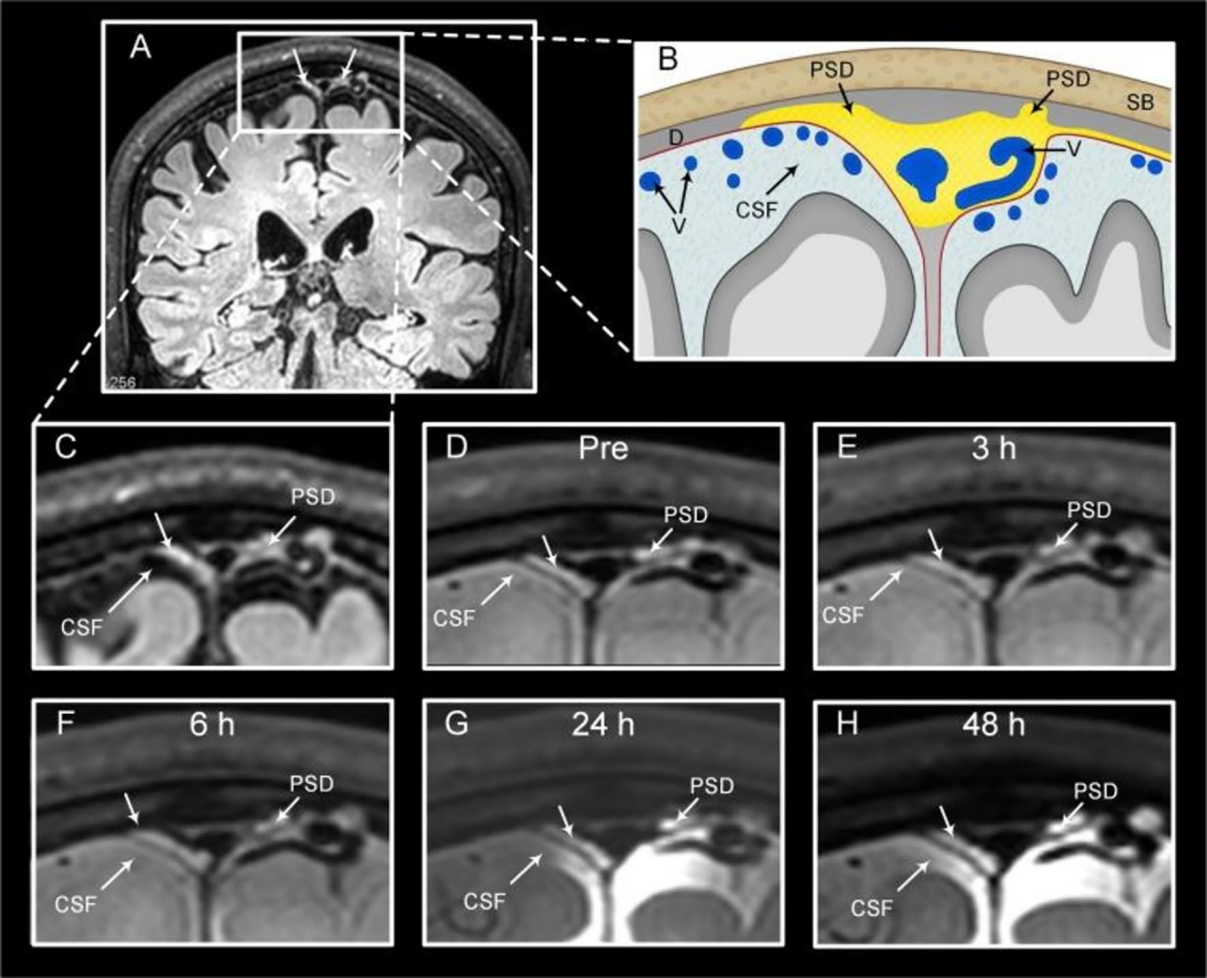
The parasagittal dura (PSD) is visible as hyperintense tissue at FLAIR (**A** and **C**). PSD (**B**) volume varies widely between subjects for unknown reasons, but has in addition to stroma been shown to contain arachnoid granulations, blood vessels, intradural channels, lateral lacunae and lymphatic vessels, the latter at micrometer scale. After injection of a CSF tracer (MRI contrast agent), PSD enhances visibly with tracer after 24 and 48 h in a diffuse fashion, but not in the shape of tubular structures. Tracer enhancement in PSD is dependent on tracer enhancement in the adjacent cerebrospinal fluid (CSF) space and is not visible before tracer has reached the CSF compartment (**D**–**F**). At 24 h (**G**) and 48 h (**H**),  From Eide PK and Ringstad (Brain Research) [28], reused with permission under the terms of the Creative Commons CC-BY license

On the contrary, a human CSF tracer study has demonstrated direct efflux from CSF into the parasagittal dura through 48 h, but could not depict signs of the tracer collecting into any tubular structures whatsoever, only diffuse enhancement [28, 49]. Peak enhancement in parasagittal dura occurred at 24 h after intrathecal tracer injection and was highly correlated with enhancement in adjacent CSF (Fig. 3). Another study using same methodology, rather interpreted a similar, diffuse enhancement pattern as “putative” meningeal lymphatic vessels [50], but without providing evidence for enhancement in tubular structures per se.

**Fig. 4:**
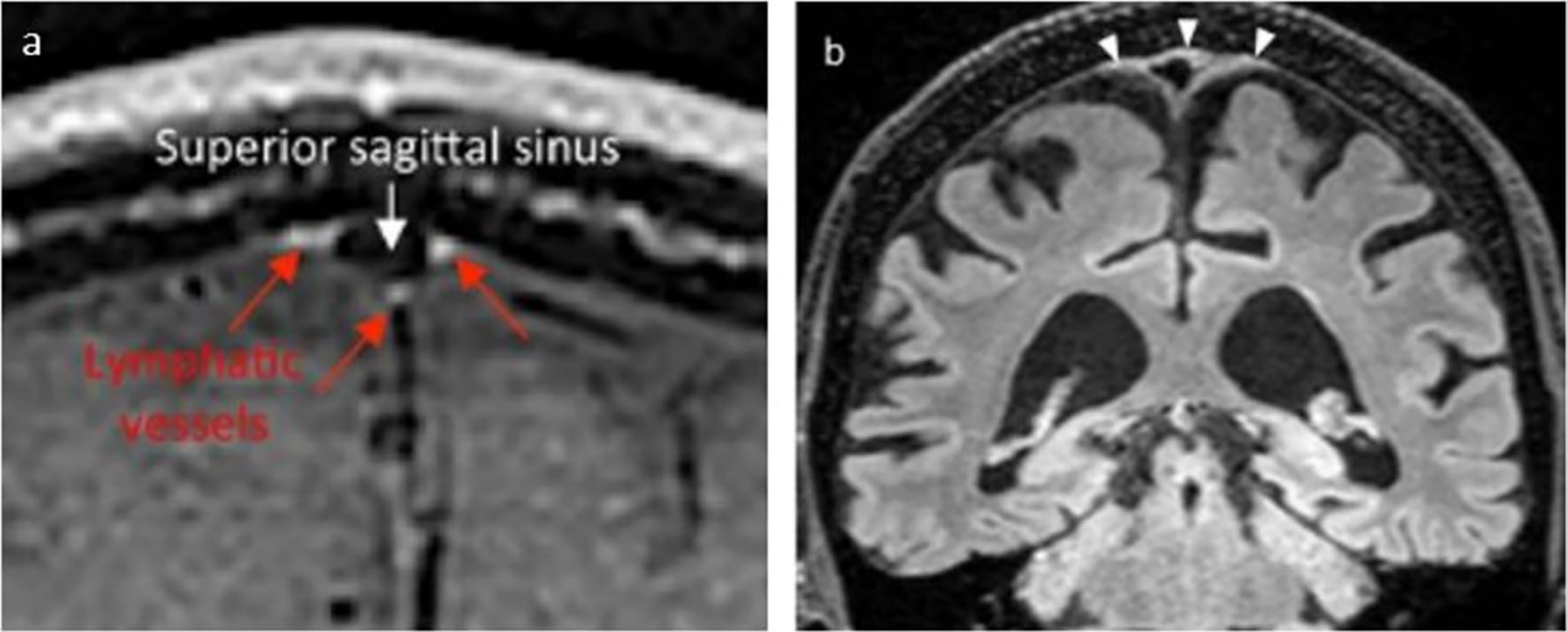
Examples of interpretation of lymphatic tissue in parasagittal dura. In **a** intravenous contrast enhancement in parasagittal dura is interpreted as lymphatic vessels (red arrows), while blood flow in superior sagittal sinus is suppressed (From Absinta et al. eLife 2017) [37]. In **b** all hyperintense FLAIR-signal in parasagittal dura (arrowheads) was accepted as lymph (From Albayram et al. Nature Communications, 2022) [54]. Figure elements are reused with permission under the Creative Commons International License

To exclude venous signals at MRI, “black blood” techniques have been applied to darken signal from slow flow within the venous vasculature [41]. However, shine-through of signal from veins with very slow flow, or artifacts from slow flowing blood in smaller vessels is one major confounding factor in the interpretation of high signal in tubular structures [51]. Whereas accompanying contrast-enhanced MR venography has further been added to subtract signals from veins that were visible at MRI [41], venous structures of microscopic size with very low flow velocity, or having caliber well below the image resolution, may not be depicted. Still, diffuse contrast enhancement in parasagittal dural regions was widely interpreted as lymph. However, blood vessels in the dura lack tight junctions and allow extravasation of solutes as large as 43 kDa [52], while for instance gadobutrol has molecular weight ~ 0.6 kDa [40]. Therefore, intravenous contrast is expected to enhance in the extra-vascular stroma of the parasagittal dura through these abundant, leaky vessels. Further sources of contrast enhancement in the parasagittal dura, whether diffuse or in shape of tubular vessels, may be derived from enrichment within networks of dural veins and capillaries, and also as proposed by Park et al. [32], enrichment within intradural channels. Absinta et al. [37] observed that a blood pool agent (gadofosveset) did not leak into the parasagittal dura, contrary to the enhancement which was observed in dura after intravenous administration of gadobutrol. Even though the enhancement they observed may be considered diffuse and at millimeter scale as revealed by 2D (Fig. 4) and not least by 3D representations, and qualitatively comparable to the enhancement in the choroid plexuses, they interpreted this as lymphatic vessels.

To this end, it can be argued that imaging of lymph by use of intravenous contrast-enhanced MRI therefore awaits further validation. Future imaging studies must be interpreted with larger care and by acknowledging existence of other sorts of tubular structures than lymphatic vessels being present in the region. While it should be expected that dural lymphatic vessels indeed exist also in the human parasagittal dura, it remains unknown whether the recently reported lymphatic vessels in human dura were within or lateral to the parasagittal dura [1]. In this regard, it should be kept in mind that the volume of parasagittal dura varies extensively between individuals [53], and the basis for this is currently not well understood. As discussed previously, meningeal lymphatic vessels within the parasagittal dura region may rather be important for immunological surveillance of the brain more than in CSF drainage [17, 19]. This is supported by human CSF tracer studies, showing that CSF flux around the upper brain convexities differs substantially between individuals [8], and with sometimes very minute amounts of CSF tracer entering from the upper SAS adjacent into the parasagittal dura [49], suggesting this is not a major efflux route. Notwithstanding this, exchange between SAS and parasagittal dura as part of neuroimmunlogical surveillance may still take place.

A method for a non-contrast approach to visualize meningeal lymph has also been suggested, where literally any hyperintense FLAIR signal outside the brain at MRI was interpreted as lymph [54]. The basis for this understanding was that increasing protein content in an external container was positively associated with FLAIR signal. Since lymph is protein rich, a hyperintense FLAIR signal was therefore accepted to derive from lymph only. As outlined above, the parasagittal dura contains many elements that can contribute to the hyperintense FLAIR signal, including small blood vessels, the lateral lacuna, intradural arachnoid granulations, and the fibrous stroma of the parasagittal dura. Interpretation of a hyperintense FLAIR signal as lymph is therefore highly presumptuous. Furthermore, even hyperintense FLAIR signals from the CSF spaces near the skull base were interpreted as ventral meningeal vessels; however, this has later been dismissed as CSF flow artifacts [55]. Finally, the authors interpreted a continuous, high FLAIR signal in the walls of neck blood vessels as lymph, too, and the continuity with similar signals at the skull base was considered to represent connections between cervical lymph nodes and the intracranial meningeal lymphatic system. A similar interpretation was reproduced in another study from the same group [56]. Again, lymph is not an exclusive source of a high FLAIR signal, and for instance connective tissue and vasa vasorum in neck vessel walls are least as likely sources. Ultimately, the reasoning behind accepting any structure with high FLAIR signal outside brain as lymph was heavily contested by two independent commentaries [55, 57]. Examples of contrast and non-contrast based interpretations of lymph at MRI are given in Fig. 4.

It remains to be determined how abundant lymphatic vessels are in human dura. Given the recent observations that fixation method extensively affects the ability to visualize lymphatic vessels in human dura [1], care should be taken when interpreting findings from formalin-fixed dura of cadavers. Accordingly, a recent study failed to identify lymphatic structures in the perisinus region, but rather tubular intradural vascular channels which stained positively with markers of lymphatic vessels that connected lateral interdural arachnoid granulations with the venous circulation [58]. In another work, it was concluded that arachnoid granulations that traverse the dura-arachnoid stroma not only serve as CSF reservoirs, but are also enriched with immune cells, supporting that perisinus tissues can represent important immune hubs at the brain surface [20]. Kutomi and Takeda also found that arachnoid granulations in pigs were lined by lymphatic endothelial cells, while dura mater in mouse was devoid of arachnoid granulations and “totally underdeveloped” compared to pig or human [59].

### Imaging evidence of glymphatic–lymphatic coupling

In humans, MRI was used for the first time to demonstrate in vivo drainage of a CSF tracer (the MRI contrast agent gadobutrol) to the lymphatic system, where the tracer enhanced subtly in cervical lymph nodes with peak after 24 h. Peak enhancement coincided with peak tracer enhancement in brain, and the finding was inferred to indicate that the tracer was cleared directly from perivascular spaces in brain to lymph after its enhancement in brain [60]. This tracer is inert, confined outside the intact BBB, and should not be expected to be cleared via other routes than the perivascular. However, later studies using the same methodology demonstrated clearly that also levels of tracer in CSF are highly associated with levels in brain at all time points through 24–48 h, well into clearance phase, suggesting that CSF clearance rate is a major determinate for brain tracer clearance. Moreover, the same CSF tracer appeared in blood with a peak merely a few hours after its intrathecal distribution, preceding peak enhancement in cervical lymph nodes by far, and also preceding its enhancement at the upper brain convexities. This sequential pattern clearly suggests that a substantial proportion of tracer was already resorbed form the spinal canal and at the skull base via meningeal lymphatic vessels. Tracer levels in CSF under the cranial vertex was a prerequisite for, and correlated with, enhancement in the parasagittal dura, clearly indicating direct efflux from CSF directly to dura, not from the brain. These human observations of efflux from CSF to meninges also resonate well with previous animal findings [2, 3], but do not exclude the possibility of an anatomically uninterrupted clearance pathway from brain to meningeal lymph.

Indeed, observations from intravenous contrast-enhanced MRI have been inferred to support a direct anatomical link between brain paravascular pathways and meningeal lymphatic vessels. Naganawa et al. demonstrated age-dependent leakage of contrast agent around the vein of Labbè [61], as well as from cortical veins at the upper convexity, into CSF at inversion recovery MRI [62]. Later, it was suggested that the contrast leaked into a space connecting the glymphatic system and meningeal lymphatics [63]. Sennfält and coworkers had a similar interpretation of this phenomenon in a recent paper [45], where they conclude that their findings suggested that interstitial fluid drainage was visible on conventional MRI and drained from brain parenchyma via cortical perivenous spaces to dural meningeal lymphatics in the parasagittal dura. As such, this drainage pathway remained separate from CSF. Observations were typically made within 30 min after intravenous contrast administration. However, this stands in sharp contradistinction to in vivo observations of CSF tracer clearance from brain, which occurs for hours and days [8]. It seems very likely that this discrepancy can be derived from an alternative cause behind the perivenous enhancement; a direct leakage of contrast agent through the venous wall. Leptomeningeal blood vessels have endothelial cells connected by tight junctions, but are not ensheathed by astrocytic foot processes, and paracellular junctions between these endothelial cells vary in tightness and can be up to 2.8 nm large [64]. Even within brain tissue, brain vessels are to some degree leaky to contrast agents, and previously established leakage constants indicate that gadolinium concentration in brain tissue after intravenous administration can be close to what is reported after intrathecal administration [65]. At arterial spin labeling MRI with ultra-long echo times, substantial and rapid exchanges of water between blood vessels and CSF at the brain surface was demonstrated [66]. Furthermore, MRI contrast agent has been shown to leak directly into CSF and the subarachnoid space at multiple locations [67–69]. Perivenous enhancement after intravenous administration was also observed in a case series after temporary opening of the blood–brain-barrier using focused ultrasound [70]. However, the phenomenon was shown to occur in merely a fraction of subjects, and it was not discussed whether the outskirts of the applied ultrasound energy field might have induced increased venous wall permeability. To this end, human MRI studies have so far only provided circumstantial evidence of an uninterrupted, compartmentalized clearance pathway from brain to lymph, and observations appear biased by expectations from theoretical frameworks.

Meningeal immunity and CSF-mediated interactions with the CNS is a flourishing research field with intense ongoing debates. Recently, it was described a previously unrecognized, fourth meningeal layer denoted subarachnoid lymphatic-like membrane (SLYM) [71]. The SLYM layer was shown to contain Prox1 + cells and to encase subarachnoid blood vessels. By its impermeability to molecules larger than 3 kD, and thereby functionally dividing the subarachnoid space into compartments, the SLYM layer would also enable for a directionalized CSF movement at the brain surface toward brain perivascular spaces, possibly linking meningeal immunity with the brain more directly. However, two later studies possibly contradicted the SLYM by claiming that Prox1 + cells were part of the arachnoid mater [72, 73]. While the possible existence of SLYM yet seems unresolved, a previous human MRI study demonstrated sign of periarterial propagation of CSF tracer within the subarachnoid space, as implicated by a SLYM layer [9]. Future human MRI studies utilizing CSF tracer and high temporal resolution could possibly resolve some of these outstanding questions.

When considering coupling between the glymphatic and lymphatic system, MRI investigators further need to be aware of a potential CSF-mediated cross-talk between the CNS and spinal dural lymphatic vessels. Spinal absorption of CSF has for long been well established to occur [74], but clearance pathways were more obscure. Recently, a continuum of meningeal and epidural lymphatic vessels in the vertebral (spinal) canal was described, with lateral exits along blood vessels and spinal nerves [75]. The spinal lymphatic vasculature was shown to interact immunologically with the spinal cord and furthermore to be particularly dense compared to the one that covers the cranial dura mater, leading to the possible explanation that spinal lymphatic vessels strongly contribute to CSF absorption [76]. A concurring study revealed that following intraventricular tracer injection in mice, tracer propagated down the spinal canal and was cleared from the subarachnoid space predominantly from the sacral spinal canal to lymphatic vessels and further to sacral and iliac lymph nodes [77]. Substantial spinal clearance was corroborated by a human CSF tracer study, where intrathecally injected MRI contrast agent at the lumbar level appeared in blood with peak concentration far earlier than peak contrast enrichment around the upper brain convexities. This constellation of findings suggests that substantial CSF clearance occurs from the spinal canal as well as the skull base. A further analysis of MRI data suggested that up to 2/3 of injected tracer in humans was resorbed via the thecal sac [78].

### Imaging evidence of coupling between CNS and bone marrow

It is remarkable that direct dural-bone marrow connections have been demonstrated physically as well as functionally, enabling for mobilization of myeloid cells into meninges and CNS parenchyma following injury and neuroinflammation [79]. Toward skull bone marrow, CSF efflux was shown for the first time in any species at human MRI [35] and was later confirmed in mice with meningitis, here also revealing that CSF and bacteria exited along perivascular spaces of dural blood vessels and through sub-millimeter skull channels [80]. The same channels directly provided for backwards migration of leukocytes to meninges. Thus, brain-derived danger signals in CSF can be sampled by regional marrow. It was recently demonstrated that the mouse skull has the most distinct transcriptomic profile compared with other bones, corroborated by the finding that skull bone marrow reflected inflammatory brain responses in patients with various neurological disorders, including inflammatory, ischemic, and neurodegenerative diseases [81]. These compelling observations in humans were accomplished by use of positron emission tomography, and it remains to be seen whether dedicated MRI could reveal bone marrow changes in response to disease.

Two-way interactions between the brain and peripheral (meningeal) and central (bone marrow) immune components have wide implications for potential mechanisms in, and modifying of, neuroinflammation, neoplastic disease and neurodegeneration [82]. Furthermore, when central and peripheral immune constituents can interact directly with the meninges and also be distributed in CSF, new hypotheses may emerge about mechanisms behind symptoms associated with common viral illnesses, including headache and sickness behavior. No human study has yet investigated CSF efflux to vertebral bone marrow, neither in the normal physiological situation nor in disease, which may eventually be accomplished with MRI contrast agents or a radioactive compound utilized as CSF tracer.

### CSF clearance as measure of meningeal lymphatic function

The acknowledgment that lymphatic vessels are the major outflow pathway of small and large CSF tracer substances, renders CSF clearance a potentially impactful marker of meningeal lymphatic clearance capacity [17]. For endogenous solutes with a role in neurodegenerative disease, one study demonstrated that impaired functional lymphatics was associated with reduced extracellular tau clearance from CNS [4]. It was proposed that the glymphatic and lymphatic systems work in tandem to clear tau to the periphery, where tau first was cleared from ISF to CSF before uptake by the dural lymphatic system. Interestingly, despite complete lack of lymphatic vessels, tau was still cleared from brain to the periphery, suggesting that other, but minor clearance routes for tau may exist. Indeed, a tau transporter over the BBB has been characterized [83]. In a Parkinson’s disease animal model, ligation of cervical lymphatic vessels aggravated accumulation of alfa-synuclein [84]. In rodents, amyloid-β is to large extent cleared from brain via other pathways than the perivascular route, including degradation by microglia and via dedicated transporters at the BBB, and merely about ¼ of amyloid-β is cleared via CSF [85, 86].

**Fig. 5:**
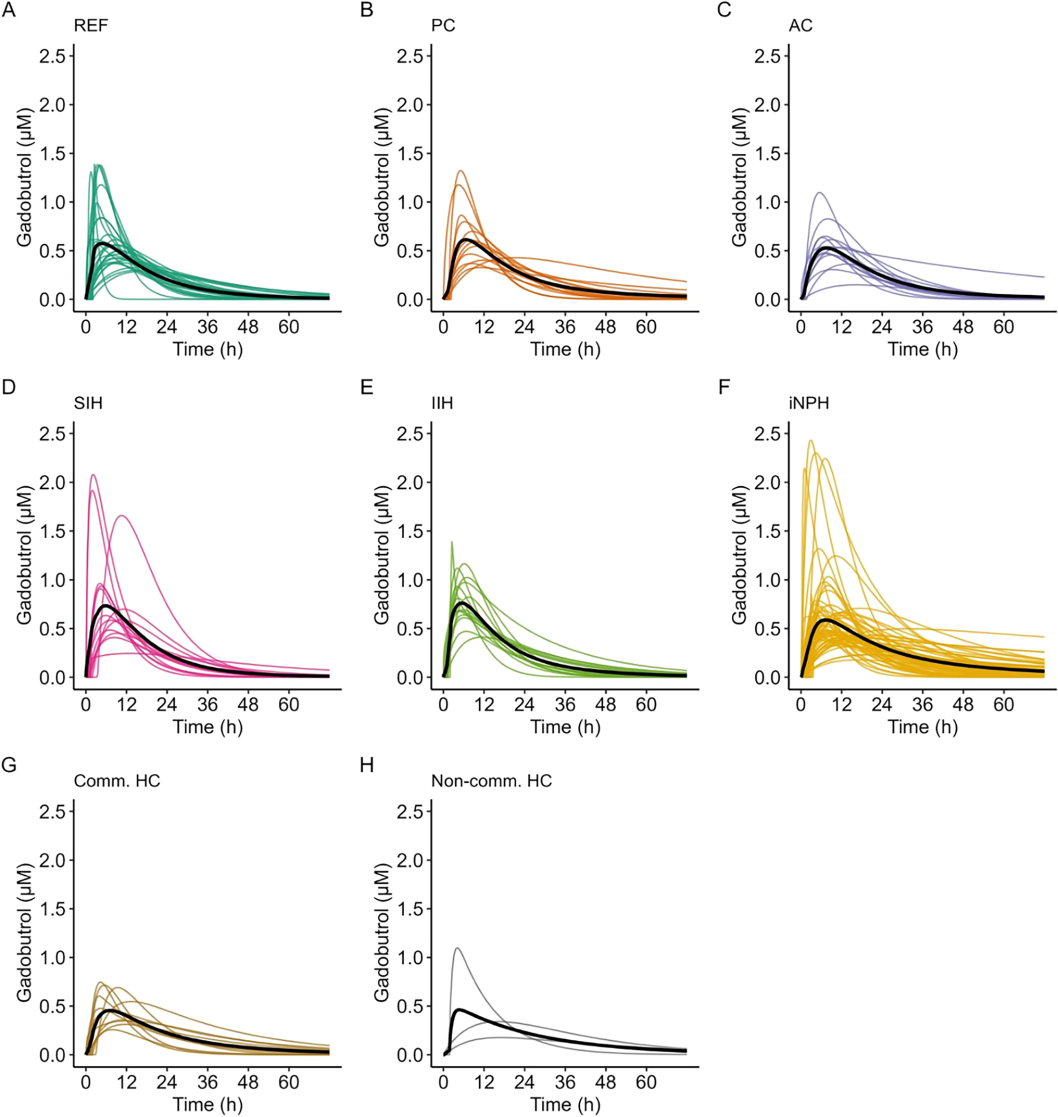
Pharmakokinetic variability in CSF-to-blood clearance of gadobutrol for reference patients (REF) (**A**), patients with pineal cysts (PC) (**B**), arachnoid cysts (AC) (**C**), spontaneous intracranial hypotension (SIH) (**D**), idiopathic intracranial hypertension (IIH) (**E**), idiopathic normal pressure hydrocephalus (iNPH) (**F**), communicating hydrocephalus (Comm HC) (**G**) and non-communicating hydrocephalus (Non-comm HC) (**H**). Black lines provide the mean predicted concentration of intrathecally injected gadobutrol in blood over time. From: Hovd MH et al. (Fluids and Barriers CNS) [92], reused with permission under the terms of the Creative Commons CC-BY license

It is therefore likely that the ability to measure CSF clearance rate would provide a suitable marker of lymphatic clearance capacity, but perhaps particularly for tau, which is deposited in brain in Alzheimer`s disease and other tauopathies. Feasibility of measuring CSF clearance rate in humans has been shown for CSF-to-blood clearance of intrathecally injected MRI contrast agent gadobutrol [87], a gadolinium–based contrast agent. Intrathecal injection of gadobutrol is “off-label” use, but the methodology can likely be converted to radiopaque contrast agents, that are generally approved for intrathecal injection, and have many molecular properties similar to gadobutrol. These features include molecular size, hydrophilic properties, the lack of binding to or reacting with proteins, and being contained outside the intact blood–brain-barrier after distribution in CSF. Thus, clearance over the blood–brain-barrier is negligible and largely anticipated to occur via meningeal lymphatic channels, both from the intracranial space and the spinal canal. Prospective safety studies of intrathecal gadobutrol in amount 0.25–0.50 mmol have shown side effects that are comparable to those reported after use of intrathecal radiopaque agents, and also after spinal punctures alone [88–90]. These observations are corroborated by the experiences from clinical use of MRI contrast agents in dose 1.0 mmol and less [91]. Remarkably, the methodology has revealed that CSF clearance not only differs between patient groups, but also among individuals within the same patient category [92] (Fig. 5). A methodological strength is that it measures overall CSF clearance and is thus independent on knowledge about particular clearance pathways, a question that is still debated [10, 93]. Being a surrogate marker of endogenous solutes of much larger size, it has yet not been validated against established biomarkers in CSF, but was associated with plasma biomarkers of neurodegeneration [94], and also with their fluctuations in plasma after sleep deprivation [95]. The methodology still lacks validation against clinical end points, such as prognostication of disease and disease progression, but can already be a marker of CSF clearance capacity for tailoring of dosage in intrathecal treatment regimens [92], since drugs in CSF must also be expected to be mainly removed along lymphatic pathways, equally to CSF itself.

## Concluding remarks

Pioneering research in animal models has positioned glymphatic brain clearance and its interplay with meningeal lymphatics at the forefront of CNS research. MRI stands out as an attractive tool to image those two systems and their coupling. MRI particularly profits from the ability to acquire a bigger perspective both anatomically and functionally within the craniospinal compartment, throughout an extended time span, and in vivo. However, both obvious and more subtle species differences mandate translation of previous animal research findings to humans. In future research, human MRI studies should liberate themselves from proposed frameworks now seemingly restraining perspectives on how imaging findings are interpreted, and better appreciate the extreme complexity of these systems. So far, interpretations of imaging findings within this young research field appear heavily biased by a desire to reproduce findings from animal studies, and very few negative studies are reported. As discussed in this review, MRI findings that have been accepted to represent lymph can least as likely be derived from other sources. Likewise, no convincing evidence has yet been provided for a direct coupling between the glymphatic system and meningeal lymphatic function, and currently, CSF is more likely to represent the intermediate step between those two systems. Meningeal lymphatic clearance function is likely the main determinate of CSF clearance rate and thereby glymphatic clearance rate along perivascular pathways. Measurements of CSF tracer clearance to blood can therefore potentially serve as surrogate marker of meningeal lymphatic clearance function. Currently, it is yet unclear to what degree glymphatic-lymphatic brain clearance contributes to total brain clearance, and how this fraction may differ between CNS regions. In this context, MR imaging of CSF flow patterns at the CNS surface emerges with new relevance, as large inter-individual variations in humans have already been revealed. Whether for instance CSF flow obstruction at the brain surface is also associated with compromise of solute clearance or immunological function, should be assessed in future research.

## Data Availability

Not applicable (review manuscript with no use of new original data).
